# Health Care Professionals’ Use of Digital Technology in the Secondary Prevention of Cardiovascular Disease in Austria: Online Survey Study

**DOI:** 10.2196/71366

**Published:** 2025-06-25

**Authors:** Luisa Lunz, Sabine Würth, Stefan Tino Kulnik

**Affiliations:** 1 Ludwig Boltzmann Institute for Digital Health and Prevention, Lindhofstrasse 22, Salzburg, 5020, Austria, 43 57255 ext 82712; 2 Department of Health Sciences, Salzburg University of Applied Sciences, Salzburg, Austria; 3 Department of Sport and Exercise Science, University of Salzburg, Salzburg, Austria

**Keywords:** barriers, cardiac rehabilitation, cross-sectional, digital health, electronic health, facilitators, mobile health, questionnaire, telerehabilitation

## Abstract

**Background:**

Advances in digital technology, such as health apps and telerehabilitation systems, offer promising treatment modalities in the secondary prevention of cardiovascular disease. However, the successful adoption of digital technology in clinical practice depends on a variety of factors. A comprehensive understanding of the influencing factors on digital technology usage in health care can support the complex implementation process of digital technology in clinical practice.

**Objective:**

The aim of this study was to identify barriers and facilitators of digital technology usage in cardiovascular disease secondary prevention from the perspective of health care professionals, and to explore whether certain characteristics of health care professionals are related to the current usage of digital technology in clinical practice.

**Methods:**

We conducted an exploratory online survey, inquiring about the perspectives and uses of digital technologies in cardiovascular disease secondary prevention. We developed an original questionnaire to address the study aim. The survey invitation was distributed among health care professionals from November 2021 to February 2022, via all cardiac rehabilitation centers, all community-based disease management services for patients with chronic heart failure, and all relevant national health care professional associations in Austria. Qualitative survey data were analyzed using thematic content analysis. Quantitative survey data were analyzed using descriptive statistics, group comparison tests, and association statistics.

**Results:**

Overall, 125 health care professionals (mean age 41, SD 11 y; n=80, 64% females) across different professions and settings, including cardiac rehabilitation phases I through IV, were recruited. General readiness for using digital technologies in the care of cardiac patients was high, but only 65 (52%) respondents reported doing so. The top 3 rated barriers to digital technology use were poor user-experience of devices and apps, lack of cost coverage, and low digital competence of patients. The top 3 rated potential application areas for digital technology were organization and appointment planning, documenting treatments, and creating personalized treatment plans. The top 3 rated facilitators for digital technology use were assurance of patient safety, assurance of patients’ privacy, and availability of technical support. Greater personal use of digital technology, younger age, and higher technology affinity of health care professionals was associated with higher readiness to use digital technology with cardiac patients.

**Conclusions:**

While there is interest in digital technology for the secondary prevention of cardiovascular disease in Austria, barriers to uptake need to be addressed. Our findings may inform the design and implementation of future digitalization projects.

## Introduction

Cardiovascular disease (CVD) remains the leading cause of death and a large contributor to loss of healthy life expectancy worldwide [1,2]. The modification of cardiovascular risk factors can have a positive influence on reducing this burden and has been a main focus of secondary prevention, for example, through exercise-based cardiac rehabilitation (CR) programs [3,4]. However, this assumes that patients can consistently adopt heart-healthy behavior changes into their daily lives, which often poses a major challenge [5].

Advances in digital technology (DT) are opening up promising ways to help patients change and self-manage their lifestyle [6]. For example, the recent European Society of Cardiology guidelines for the management of chronic coronary syndromes now include a class 1A recommendation for mobile health interventions to improve patient adherence to healthy lifestyles and medical therapy [7]. Such interventions, incorporating text messaging, smartphone apps, web-based content, and wearable devices, have been shown to support patients’ healthy behaviors including medication adherence [8], exercise habits [8-11], and diet [9]. Demonstrated effects on clinical outcomes are improved blood pressure control [8,10], increased exercise capacity [12], reduced waist circumference [10], reduced low-density lipoprotein levels [9,10], decreased incidence of major adverse cardiovascular events [12], and improved quality of life [12].

Furthermore, DT has facilitated the provision of telerehabilitation, that is, home-based CR programs delivered remotely by CR professionals, which could increase access to a structured and supervised exercise-based CR program for patients who are unable or unwilling to attend a center-based CR program [13]. High-level evidence shows that telerehabilitation compared to center-based CR offers equivalent effects on patient outcomes in terms of medication adherence, smoking behavior, physiological risk factors, depression, functional capacity, exercise behavior, cardiac-related hospitalization, and quality of life [14,15].

While the scientific evidence for DT in the secondary prevention of CVD is strong, its implementation in real-life practice often lags behind [16]. The successful adoption of DT in clinical practice depends on a variety of factors, for instance, on the technology itself, its promised benefits for patients, organizational and systematic factors, as well as the characteristics, attitudes and experiences of the various user-groups (eg, patients, caregivers, and health care professionals [HCPs]) [16,17]. The scoping review by Whitelaw at al [18], for example, lists the following commonly reported clinician-level barriers to uptake of DT in cardiovascular care: increased work and responsibilities, unreliable technologies, lack of evidence supporting the use of technology, and lack of integration with medical records. The most commonly described clinician-level facilitators were approval and organizational support from senior management and improved efficiency through DT [18]. Because the organization, structure, and funding of health care systems can differ considerably from country to country, a comprehensive understanding of the influencing factors on DT usage in a national health care context can support the complex implementation process of DT in clinical practice [17].

The aim of this study was to identify barriers and facilitators of DT usage in CVD secondary prevention from the perspectives of HCPs in Austria. Specifically, we sought to identify HCPs’ attitudes toward DT usage, and to explore whether certain HCP characteristics (affinity for DT, personal use of DT, age, physical activity [PA] behavior, and professional background) are related to the current usage of DT in clinical practice.

## Methods

### Overview

We conducted a cross-sectional online survey among HCPs working in the secondary prevention of CVD in Austria. In the reporting of this study, we adhere to the Checklist for Reporting Results of Internet E-Surveys (CHERRIES) [19].

### Setting and Participants

Our survey addressed settings for the secondary prevention of CVD in Austria, including general practitioner and cardiologist practices, outpatient clinics, community-based disease management programs for patients with chronic heart failure, and the CR pathway. In Austria, the latter comprises 4 phases: the acute hospital stay (phase I), medically supervised in- or outpatient rehabilitation programs of up to 6 weeks duration (phase II), medically supervised outpatient rehabilitation programs of 6‐12 months duration with weekly or less frequent sessions (phase III), and patients’ life-long independent secondary prevention behavior and self-management (phase IV) [20]. We invited qualified HCPs from any relevant professional background (including nurses, physicians, sport scientists, physiotherapists, psychologists, and dietitians) who were working in any of these settings. Unemployed HCPs and retirees were excluded from the survey.

### Recruitment

Recruitment took place between November 1, 2021, and February 20, 2022. Email invitations with an open link to the online questionnaire were sent to the medical and nursing directors of all CR centers (at the time 13 inpatient and 21 outpatient centers); to all 3 community-based disease management services for patients with chronic heart failure; and to the boards of all relevant HCP associations (cardiology, dietetics, nursing, nutrition science, occupational therapy, physiotherapy, psychology, social work, and sports science) in Austria. The addressees were asked to forward the survey invitation to all employees or members of their organizations.

### Sample Size

This exploratory survey recruited a convenience sample, and no prospective sample size calculation was conducted. Based on response rates from previous online surveys among HCPs in Austria that used similar recruitment strategies, we expected to achieve a sample size of 100 to 200 respondents.

### Survey Instrument Development

Because no valid survey instrument existed that aligned with the study aims, an original questionnaire was designed, implemented in LimeSurvey (version 3.25.21+210407) and piloted. The questionnaire’s content was developed based on qualitative data (interviews and focus group) from 7 CR professionals and literature on obstacles and potential application areas for DT in health care [21-23]. Then, the questionnaire was piloted using cognitive interviewing with 8 HCPs from different professional backgrounds who were representative of the target sample. The questionnaire was iterated and revised twice to optimize comprehensibility, usability, completion rate, and completion time. The development process supports content and construct validity of the survey instrument, but we were unable to perform psychometric assessments of construct validity (eg, convergent validity) due to the lack of suitable validated alternative measures.

### Questionnaire

The questionnaire consisted of 42 items divided into 10 sections. Items were formulated as multiple-choice questions, Likert scale items [24] and open questions with free-text answers. The estimated completion time was 20 minutes. The full questionnaire in its original German version is available at the Open Science Framework platform [25], and an English translation is given in Multimedia Appendix 1. In summary, the questionnaire covered the following content:

- Professional profile (5 items).
- PA behavior (meeting the World Health Organization [WHO] recommendations [26]; 4 items).
- Affinity for DT (2 polarizing questions selected from the TA-EG questionnaire [27]; 1 item).
- Personal use of DT (types of digital devices used—in particular devices relating to PA, 3 items).
- Use of DT at work in cardiovascular care (recommending the use of DT to patients, reasons for recommending or not recommending DT to patients, types of DT used with patients or for patient care, reasons for non-use of DT, DT used for certain patient groups only, past use of DT and reasons for discontinuing, knowledge of DT used in cardiovascular care by other HCPs or in another setting; 12 items).
- Readiness for using DT in their work (1 item).
- Perceived barriers to using DT in cardiovascular care (rating of 20 potential barriers, 1 open question; 5 items).
- Potential application areas for DT in cardiovascular care (rating of 17 potential application areas, 1 open question; 3 items).
- Factors influencing the decision to use or not use DT (rating of 22 potential influencing factors, 1 open question; 4 items).
- Demographic information (gender, age, highest education level, professional qualification; 4 items).

### Ethical Considerations

The study was reviewed by the research ethics committee of the University of Salzburg and received favorable ethical opinion (reference GZ 21/2021). Survey respondents were presented with information about the study and contact details of the study team on the first page of the online questionnaire. Respondents had to first confirm their informed consent in the online questionnaire, before anonymously completing the survey questions. A voluntary prize draw of three smart watches and fitness trackers, worth US $200 each, served as incentive for participation. To maintain anonymity of survey responses, email addresses required for prize notification were collected separate from the survey responses.

### Data Cleaning

Verification of data completeness was not necessary, as only fully completed surveys were saved to the platform. Individual respondents’ completion time was reviewed to reduce the likelihood of dishonest answers (eg, overly fast completion time).

### Data Analysis

Qualitative data from free-text answers were analyzed using thematic content analysis [28]. Quantitative data were analyzed descriptively. Group comparisons were conducted using *t* test, Man-Whitney *U* test and Kruskal-Wallis test (2-tailed, alpha=.05). We calculated associations between HCP’s PA behavior and their personal use of DT, and HCP’s age, sex, and professional background and their affinity for DT. To explore whether certain HCP characteristics were related to the current use of DT in clinical practice, we calculated bivariate association statistics between the predictor variables affinity for DT, personal use of DT, age, sex, PA behavior, and professional background, and the outcome variables DT recommendation behavior, DT implementation behavior and readiness to use DT in practice, applying the appropriate statistical tests (*χ*^2^ test, binary logistic regression, and Spearman correlation coefficient). All statistical analyses were performed in SPSS software (version 22.0, IBM) and without correction for multiple testing due to their purely exploratory nature.

## Results

The survey recruited 125 participants. Respondent characteristics are given in Table 1.

**Table 1.** Respondent characteristics.

Characteristic	Sample (N=125), n (%)
Age (years), mean (SD)	41 (11)
Sex	
Female	80 (64)
Male	38 (30)
Nonbinary	1 (1)
Not disclosed	6 (5)
Education	
Compulsory schooling, apprenticeship	8 (7)
A-levels or equivalent professional education	24 (19)
University	93 (74)
Professional qualification^a^	
Nursing	40 (32)
Medicine	25 (20)
Sports science	21 (17)
Other	17 (14)
Physiotherapy	13 (10)
Psychology	13 (10)
Dietetics	10 (8)
Medical assistant	3 (2)
Administration	1 (1)
Social work	1 (1)
Clinical remit^a^	
Nursing care	40 (32)
Medical care	25 (20)
Medical training therapy	23 (18)
Administration	19 (15)
Social work	19 (15)
Physiotherapy	12 (10)
Nutrition advice	11 (9)
Psychological care	9 (7)
Smoking cessation	7 (6)
Other	5 (4)
Sports science	2 (2)
Setting^a^	
Outpatient rehabilitation center	43 (34)
Inpatient rehabilitation center	41 (33)
Acute hospital – inpatients	29 (23)
Private practice	12 (10)
Acute hospital – outpatients	8 (6)
Patient home visits	5 (4)
Other	4 (3)
Non–health care setting	1 (1)
Cardiac rehabilitation phase^a^	
Phase I	27 (22)
Phase II	79 (63)
Phase III	44 (35)
Phase IV	33 (26)
Community-based disease management program for patients with chronic heart failure	8 (6)
Other	5 (4)

a Multiple answers possible.

### HCPs’ Affinity for DT, Personal Use of DT and PA behavior

Most HCPs rated themselves tech-savvy (median 2, IQR 2-3; on a 5-point Likert scale from 1 [“very tech-savvy”] to 5 [“not at all tech-savvy”]). Only 5 (4%) respondents reported no personal use of DT, with others using smartphones (n=114, 91%), wrist-worn heart rate sensors (n=51, 41%), smartwatches (n=35, 28%), step counters (n=33, 26%), watches with chest strap for heart rate measurement (n=27, 22%), and digital devices for measuring physical performance (n=12, 10%). Older HCPs had lower affinity for use of DT (rho=0.24, 95% CI 0.06‐0.41; *P*=.006). There were no significant differences in affinity according to sex or professional group. A total of 54 (43%) respondents reported meeting the WHO PA recommendations for adults (≥150 minutes per week of moderate or ≥75 minutes per week of vigorous intensity endurance-type PA; and ≥2 times per week muscle strengthening activities) [26], with 56 (45%) reporting recording, planning or sharing their PA using DT. Binary logistic regression revealed a higher likelihood of personal use of DT (in particular devices with PA-related functionalities) for those who met the PA recommendations, as compared to those who did not (OR 2.8, 95% CI 1.4‐5.9; *P*=.005).

### Recommendation and Usage of DT in Practice

Respondents’ subjective readiness to use DT in clinical practice was high (median 2, IQR 1-2; on a 5-point Likert scale from 1 [“very inclined”] to 5 [“very opposed”]). Overall, 88 (70%) respondents reported that they currently recommended the use of DT to their CVD patients. A total of 80 respondents listed their most common recommendations in free text answers. These were for smartwatches and heart rate monitors (n=47, 59%), apps (n=36, 45%), and step counters (n=16, 20%), primarily for aspects of training control, heart rate monitoring, and recording or visualizing of vital signs, training and PA behavior. A total of 65 (52%) HCPs reported currently using DT as part of their clinical practice with CVD patients, including chest straps (n=32, 49%) and wrist watches (n=17, 26%) for heart rate measurement, apps (n=21, 32%), online information (n=12, 18%), step counters (n=12, 18%), smartwatches (n=11, 17%), and activity trackers (n=10, 15%). The most used apps were HerzMobil, heartfish, and RehaApp. HerzMobil (Landesinstitut für Integrierte Versorgung Tirol, Innsbruck, Austria) is part of a telemonitoring system in conjunction with Bluetooth-enabled blood pressure devices and scales [29]. The system was offered by one regional heart failure disease management service in Austria. The cost of HerzMobil was covered by a regional public healthcare fund. heartfish (heartfish GmbH, Vienna, Austria) is an app that aims to support motivation and adherence with exercise therapy in patients with CVD, cancer, and other conditions [30]. heartfish was in use at several outpatient CR centers in Austria. The basic version of the app was made available to patients for free, with the option of a paid subscription for extended functionalities. RehaApp (Pensionsversicherungsanstalt, Vienna, Austria) is an app to support self-monitoring of blood pressure, body weight, medication, and PA adherence following an inpatient rehabilitation stay. The app was in use as part of a clinical trial at inpatient CR centers in Austria.

### Reasons for Non-Recommendation and Non-Usage of DT in Practice

Reasons given in free text for non-recommendation and non-usage of DT in practice are listed in Table 2. The most common reasons for not recommending DT to CVD patients were the feeling of it not being within one’s area of responsibilities or allocated tasks, lacking technical skills, as well as concerns over the patient becoming too dependent on DT or reducing their sense of body awareness. The most frequently given reason for not using DT in practice was lack of opportunity or possibility to do so, followed by the patient’s age, not feeling responsible for it, lack of familiarity with suitable options, and not having enough time.

**Table 2.** Reasons for non-recommendation and non-usage of digital technologies in practice.

Question	Responses		
	Relating to the patient	Relating to the health care professional	Relating to the physical and social environment
If you can think of any specific reasons why you do not recommend digital technologies to your patients, please describe them here (n=21)	Concerns regarding loss of body awareness and risk of dependence on digital technologies (n=4) Too overwhelming (n=3) Age (n=2) Pressure to perform (n=2) Compliance (n=1)	Not within one’s own area of responsibility or tasks (n=5) Lack of own technical competence (n=4) Lack of exposure to possible digital technology (n=3) Lack of time (n=2) Not interested in advertising products (n=1)	—^a^
If you can think of any specific reasons why you do not implement digital technologies into your clinical practice, please describe them here (n=30)	Age (n=5) Patients already use digital tools independently (n=1)	Perceived as outside one’s responsibility (n=3) Lack of familiarity with practical, appropriate, ad-free options (n=3) Lack of time (n=3) Focus on personal coaching (n=1) Lack of communication skills (n=1)	Lack of opportunity or possibility (n=11) Poor internet connection (n=1) Lack of implementation in the work process (n=1)

a Not applicable.

### Barriers

**Figure 1.**
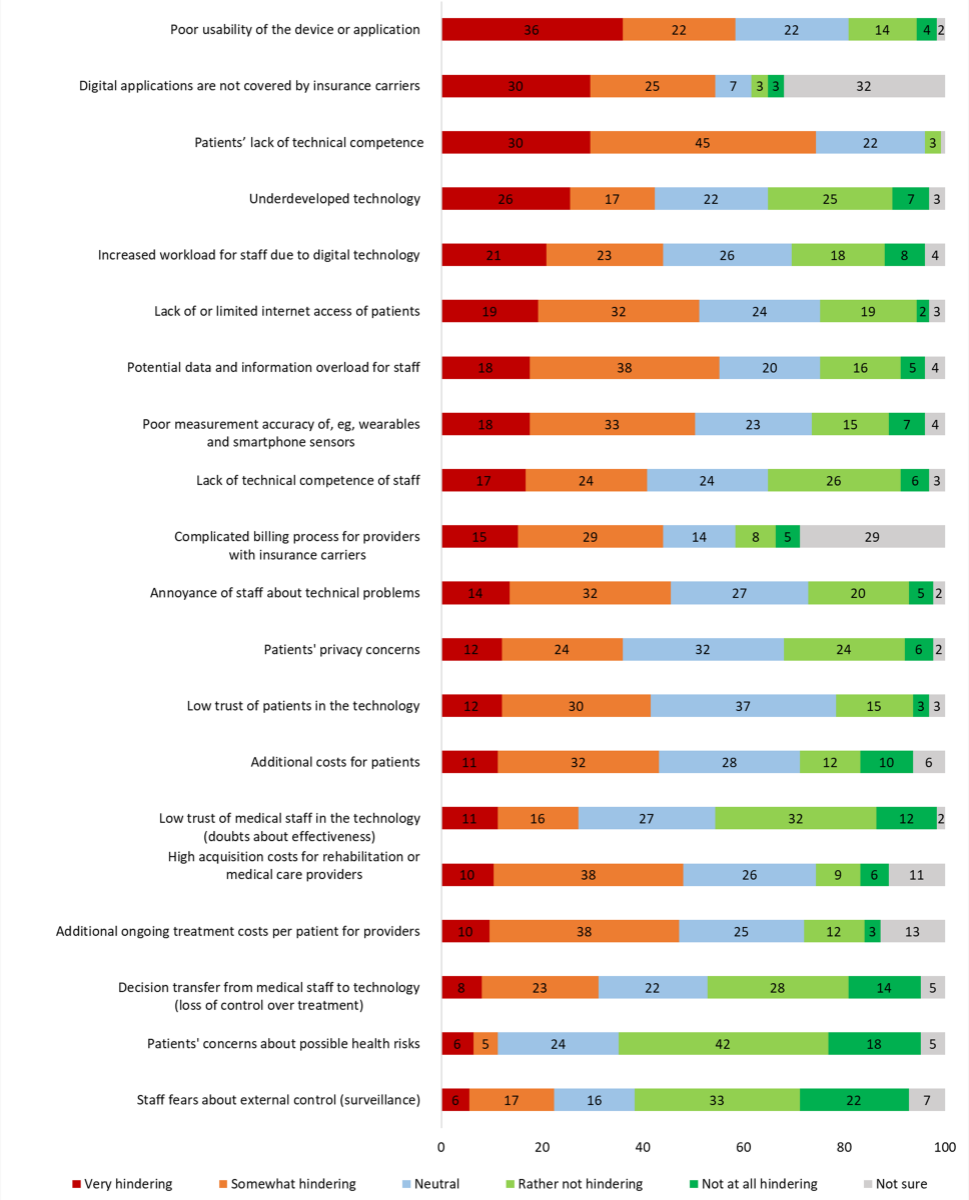
Barriers to the use of digital technologies in the secondary prevention of cardiovascular disease. Respondents (N=125) rated each potential barrier on a 5-point Likert scale. The percentages for each response category are shown.

The top 5 rated barriers (answer “very hindering”) of using DT in practice were poor usability, lack of reimbursement from insurance carriers, patients’ lack of technical competence, underdeveloped technology, and fear of increased workload for staff (see Figure 1). The latter point was reiterated 8 times in the free-text answers.

### Potential Areas of Application

**Figure 2.**
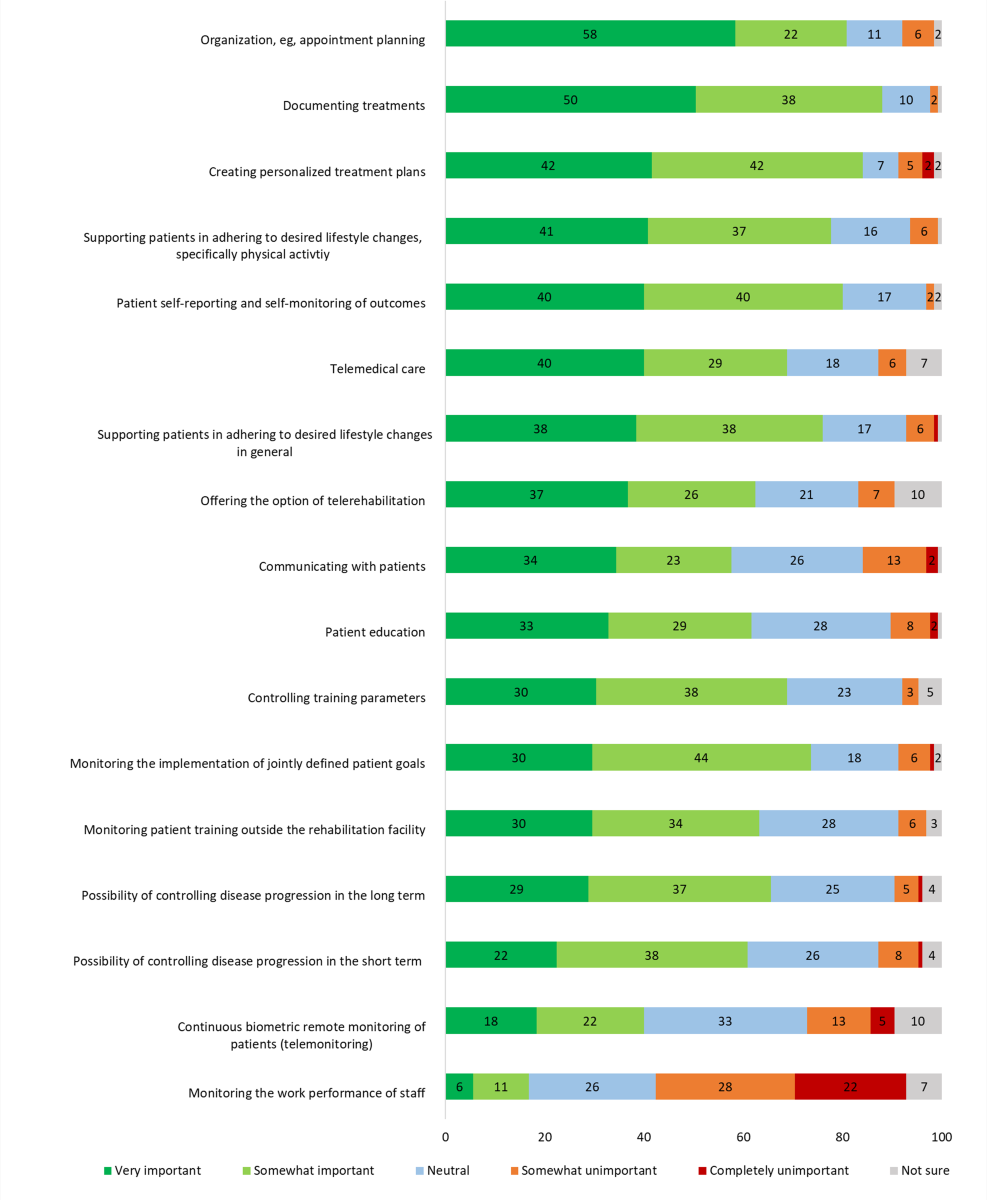
Potential important application areas of digital technologies in the secondary prevention of cardiovascular disease. Respondents (N=125) rated each potential application area on a 5-point Likert scale. The percentages for each response category are shown.

The application areas for DT that were perceived as most relevant (marked “very important”) were for organization, documentation of measurements, creating personalized treatment plans, supporting patients in their adherence to PA lifestyle change, and patient self-reporting of outcomes (see Figure 2).

### Factors Influencing the Decision to Use DT

**Figure 3.**
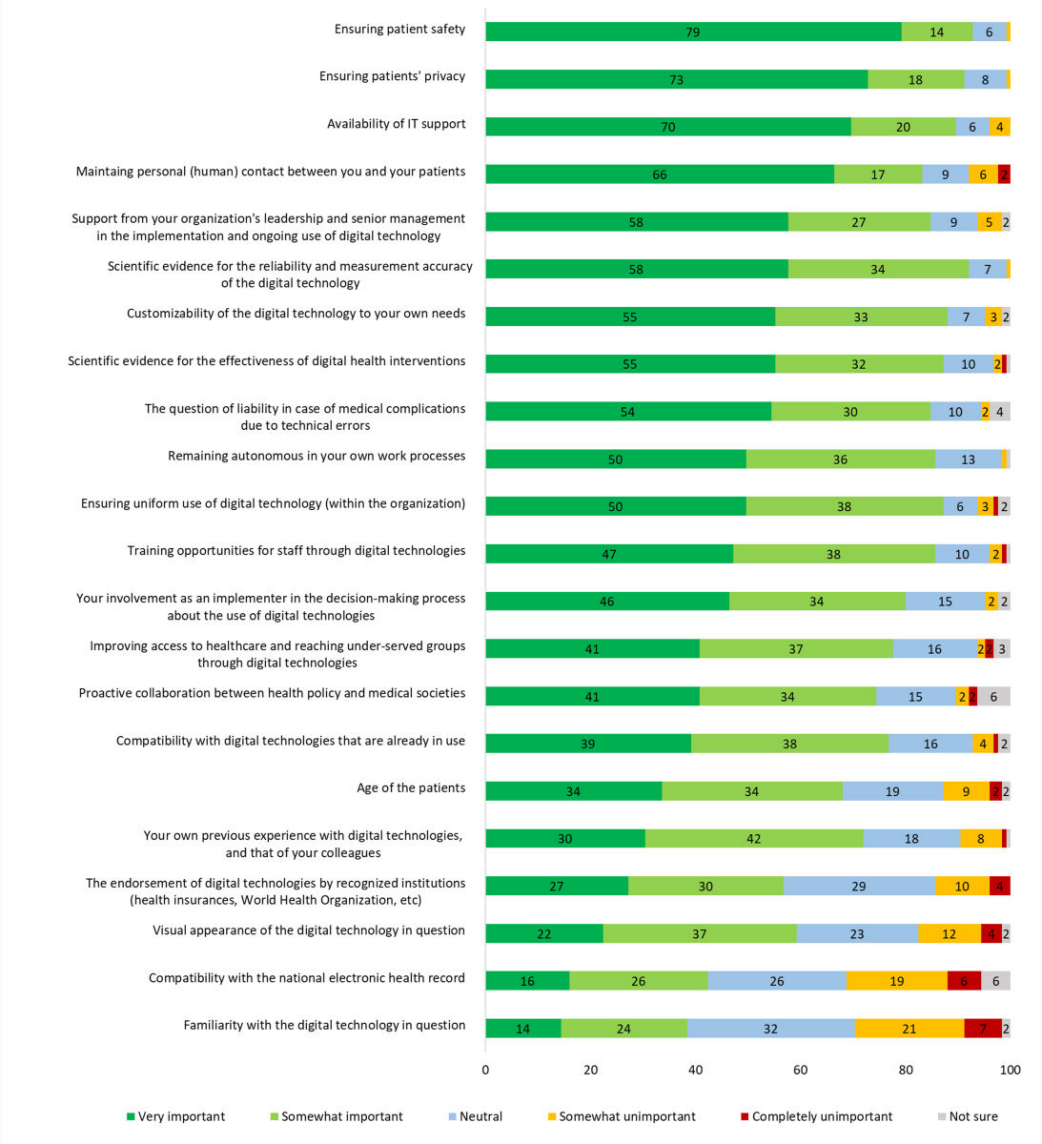
Factors that influence the decision to use or not use digital technologies in the secondary prevention of cardiovascular disease. Respondents (N=125) rated each potential factor on a 5-point Likert scale. The percentages for each response category are shown.

The highest rated influencing factors (marked “very important”) for using DT in practice were assurance of patient safety and privacy, availability of technical support, and the maintenance of personal contact between HCPs and their patients (see Figure 3).

### Recommending DT to Patients

HCPs’ inclination to recommend DTs to their CVD patients was not related to HCPs’ own PA behavior or their personal use of DT, nor did the mean age of those who recommended DT (41, SD 12 y) versus those who did not (40, SD 10 y) differ significantly. In addition, affinity for DT did not significantly differ for recommenders and non-recommenders, with a median of 2 (IQR 1.5-3; “rather tech-savvy”) in both groups. However, the likelihood of recommending DTs was significantly higher among medical doctors compared to other professions (OR 7.3, 95% CI 1.4‐38.3; *P*=.02).

### Implementing DT in Clinical Practice With Patients

The use of DT in clinical practice was not statistically related to HCP’s own PA behavior, their personal use of DT, their sex or professional and academic background, nor did the mean age of users (42, SD 12 y) and non-users (39, SD 10 y) or affinity for DT across users and non-users differ significantly.

### Readiness to Use DT in Practice

HCP’s subjective readiness to use DT in practice was not related to their own PA behavior or professional and academic background. However, descriptively, sport scientists reported the highest readiness to use DT with a median of 1.5 (IQR 1-2), and psychologists the lowest with a median of 2.5 (IQR 2-3). Respondents who personally used DT demonstrated a significantly higher readiness to do so in clinical practice, as compared to those who did not (mean 1.7 SD 0.8 vs 2.2 SD 1.0, respectively; *P*<.001). Furthermore, older HCPs felt less ready to use DTs in practice (r_s_=0.22, 95% CI 0.04‐0.39; *P*=.01), and those with higher affinity for DT felt more ready to use DTs in practice (r_s_=0.47, 95% CI 0.31‐0.60; *P*<.001).

## Discussion

### Principal Findings

We found that respondents’ readiness and attitudes toward using DT in the secondary prevention of CVD were generally positive. However, in comparison, their current usage of DT in practice was relatively low at just over 50% across all professions, and particularly low among dieticians, nurses, physiotherapists, and psychologists, of whom less than half reported implementing DT.

HCPs’ age was not significantly related to the usage or recommendation of DT in clinical practice, but older age was associated with lower readiness for DT implementation and lower affinity for DT. The threat of ageism to successful digital engagement is increasingly being highlighted, and authors call for awareness-raising and training to achieve a positive framing of older age in the digital world [31]. As such, HCPs’ age may not constitute a major obstacle in the usage of DT but should be considered in the training and integration phases of DT in clinical practice. The successful implementation of new DT requires organizational and collegial support [32]. For instance, specialized training options offered to older HCPs might increase readiness to use DT, and thus, contribute to a successful implementation of DT in clinical practice. Furthermore, peers who act as implementation “champions” can assist in building positive experiences of digitalization for their colleagues. The concept of implementation champions stems from implementation science and describes a role occupied by people who are internal to an organization, have an intrinsic interest to implementing a change, and are committed to drive implementation forward [33]. Our data describe a profile of younger, more physically active HCPs with greater affinity and personal use of DT and higher readiness to use DT with patients. Such individuals, among others, could be enlisted to act as implementation champions and peer supporters for colleagues.

In terms of PA, HCPs who met the WHO PA recommendations were nearly 3 times as likely to personally use DT than those who did not. Thus, it is plausible that PA increases with DT usage, as studies report increased daily active minutes and steps through smartphone app or wearable usage [34]. On the other hand, physically active respondents may simply be more inclined to use DT to manage or track their PA, which would be reflective of the types of fitness apps that respondents in our sample most frequently used in their private lives (ie, Strava, Garmin Connect, and Polar Flow). Interestingly, the personal usage of smartwatches for heart rate measurement was less frequently reported by respondents, with just slightly over half reporting so.

In HCPs’ work-related use of DT, heart rate monitors, smartwatches, and apps were the most frequently used and recommended devices. Regarding the named apps, it is noticeable that these target multiple cardiovascular risk factors and clinical parameters. Apps that focus on single health behaviors (eg, smoking cessation) or more specific clinical issues (eg, mental health) were not listed. There is some evidence to suggest that digital health interventions that target multiple health behaviors or CVD risk factors could be more potent, for example, in the systematic review by Akinosun et al [9]. But apps that focus on single health behaviors or cross-cutting topics such as mental health could be equally relevant and appropriate in CVD secondary prevention [35], and such apps are currently more widely available than CVD-specific mobile health solutions, for example, in the German directory of approved and reimbursed digital health applications [36]. The prevalence of chest straps for heart rate measurement was higher than wrist-worn sensors, possibly due to chest straps having been established for longer in CR. But it may also reflect that many wrist-worn sensors are still less accurate than chest straps for measuring heart rate, which would correspond with the eighth-rated barrier in our survey [37].

With regard to potential application areas for DT in the secondary prevention of CVD, HCPs perceived organization and appointment scheduling as the most relevant, especially in the early phases of CR when regular contact and scheduling is required, followed by documentation of treatments. For instance, a uniform, digital system could be helpful in seamlessly tracking measurements. At home, the use of an app could allow patients to visualize results, better inform themselves, and monitor their own parameters. Creating personalized treatment plans and supporting patients with behavioral changes (specifically PA behavior but also desired lifestyle changes in general) were other highly ranked potential application areas, which mirrors other studies of HCP’s perceptions of digital health in cardiac care [38]. A further highly ranked potential application area concerns the provision of remote care, including telemedical care in the sense of remote individual consultations via video or telephone calls as well as offering structured and supervised CR programs via telerehabilitation formats in addition to center-based in- or outpatient CR. While the COVID-19 pandemic has to some extent forced HCPs to establish remote formats for individual consultations, telerehabilitation options for phase II or III CR programs are still lacking in Austria to date, despite their potential to increase the reach and uptake of CR among patients who do not engage with center-based rehabilitation [13].

The highest-rated barriers to DT usage in our survey included poor usability, increased workload for staff, patient age, and lack of cost coverage, which corresponds with commonly reported barriers in the literature, for example, in the scoping review of 29 primary studies by Whitelaw et al [18]. In our qualitative survey responses, concerns over patients’ dependence on DT was the most frequently listed patient-related barrier, corroborating some smaller qualitative studies, which have also raised this point. For instance, Attig and Franke [39] reported decreased PA motivation when commonly worn fitness trackers were not available for users, for example, when the device had been forgotten or its battery was empty; and other qualitative studies of CR patients have observed patients’ own concerns about dependence on DT [40]. However, the number of studies reporting positive effects of fitness tracking on users’ motivation to be physically active [10,12] suggests that, while a risk of dependence should be taken into account, the increased motivation elicited by DT may outweigh the potential consequences of dependence.

Poor usability and increased workload were also reported barriers in a recent qualitative study that evaluated the implementation of a digital CR intervention [41]. Poor usability and increased workload go hand-in-hand, as poor usability increases workload demands. As such, well-designed and optimized DT can aide in overcoming these barriers. User-centered co-design constitutes a methodological cornerstone to achieve this and is gradually finding increasing application in the development of interventions for the secondary prevention of CVD [42].

Old age or perception of age-related barriers, such as DT not being suitable for older patients, were reported hindrances of DT usage in clinical practice. As this can lead to perpetuation of negative ageist stereotypes and exclusion of older patients from digital health interventions [31], consideration of ways to facilitate older patients’ participation in DT usage is needed. In addition to individual-level strategies such as communicating personal benefits of DT for older people and offering age-tailored instructional materials and training in DT use to patients [43], meso-level strategies are required, including changing the negative discourse on aging, and inclusion and partnership with older people in the design of DT and digital health care services [31,44]. Rather than gatekeeping the provision of DT according to the perceived digital competency of patients, HCPs may find that many individuals who are less familiar with DT are able to engage with digital health interventions with minimal assistance [45].

Finally, the lack of cost coverage by insurance providers hindered HCPs from using DT. Although, there is good scientific evidence of the health-promoting effects of DT in the secondary prevention of CVD [46,47], there is currently still no established reimbursement system for digital health interventions in Austria and many other European countries. Austria’s journey towards embracing digital health started 2 decades ago, with the decision to introduce a national electronic health record system [48]. But concrete efforts towards a reimbursement system for digital health interventions have only started in 2023, concurrently with the development of the first national eHealth strategy for Austria [49]. While other European countries, notably Belgium, Germany, and France, have been more proactive in setting up transparent reimbursement systems for digital health interventions [50], Austria plans to create a process by 2026, which is expected to act as a catalyst for the implementation of DT in clinical practice. In this, it will be important to guard against inherent inequity and widening of the digital divide, which is driven not only by the direct costs of DT to HCPs and patients (eg, licenses and subscriptions), but also by structural and socioeconomic disadvantage among the population, including the lack of network infrastructure (internet broadband access, data allowance), the affordability of smartphones and computers, and limited digital literacy [51,52]. In Austria’s publicly funded health care system with near-universal coverage [53], direct costs of DT can be expected to have lesser impact on inequity, but structural and socioeconomic disadvantage alongside collateral and hidden costs for enabling inclusive digital health, such as the provision of digital skills training for patients, need to be taken into account.

### Limitations

Our survey was limited by the self-selected nature of the sample, leading to possible selection bias towards individuals with interest in the topic, for example, those with greater affinity and more positive attitudes towards DT. This likely accounts for the high levels of affinity for DT and subjective readiness to use DT in clinical practice among the sample. We acknowledge that the questionnaire did not capture respondents’ responsibility or role with regard to DT in clinical practice, that is, whether they were a prescriber or they executed a prescription. Although we were able to recruit respondents across the different professions involved in CVD secondary prevention in Austria, our findings are to be interpreted as exploratory rather than representative. The lack of a prospective sample size calculation is acknowledged.

### Conclusion

We conducted the first nationwide Austrian survey to capture HCPs’ perspectives and use of DT in CVD secondary prevention. We describe the currently prevalent types of digital health interventions and digital devices and give insight into HCPs’ perspectives on relevant application areas, barriers, and facilitators for DT in CVD secondary prevention. These findings can sensitize digital intervention developers, researchers, and implementers to HCPs’ needs and wants with regard to DT, thereby contributing to the successful design and implementation of digitalization projects in CVD secondary prevention.

## Supplementary material

10.2196/71366 Multimedia Appendix 1 English translation of the online questionnaire.

## Abbreviations

CR: cardiac rehabilitation
CVD: cardiovascular disease
DT: digital technology
HCP: health care professional
PA: physical activity
WHO: World Health Organization
