# Supplementary material for: Health Care Professionals’ Use of Digital Technology in the Secondary Prevention of Cardiovascular Disease in Austria: Online Survey Study
Source: JMIR Cardio. 2025 Jun 25;9:e71366. doi: 10.2196/71366 (PMC12221187; doi:10.2196/71366)
Supplement: Multimedia Appendix 1 [file cardio-v9-e71366-s001.docx]

**Supplementary file 1.** English translation of the online questionnaire

This English translation is for the information of English-speaking readers only. It is not a validated English version of the original questionnaire.

The questionnaire in its original German version is available at the Open Science Framework platform <https://osf.io/> (DOI: 10.17605/OSF.IO/N9JZ6).

**Part A: Work with Cardiology Patients**

**A1.** First, we would like to ask you for some information about your work with cardiology patients. Please select the answers that apply to you.

How many years have you been working with cardiology patients?

**A2.** In which phase of rehabilitation or service are you currently working with cardiology patients?

(Multiple answers possible)

- Cardiological rehabilitation phase I (acute hospital)
- Cardiological rehabilitation phase II (4-6 weeks, outpatient or inpatient rehabilitation)
- Cardiological rehabilitation phase III (6-12 months, outpatient rehabilitation)
- Cardiological rehabilitation phase IV (lifelong, self-directed, heart-healthy behavior in the patient’s daily life)
- Services for optimizing disease management (e.g., KardioMobil in Salzburg)
- Other: ___

**A3.** In which area of care for cardiology patients are you involved?
(Multiple answers possible)

- Medical care
- Nutritional counseling
- Health and nursing care
- Medical exercise therapy
- Organization/administration
- Physiotherapeutic care
- Psychological care
- Smoking cessation
- Social work
- Sports science support
- Other: ___

**A4.** In which setting are you active in the care of cardiology patients?
(Multiple answers possible)

- Acute hospital – inpatient area
- Acute hospital – outpatient clinic
- Inpatient rehabilitation facility
- Outpatient rehabilitation facility
- Private practice/freelance
- Non-medical settings (e.g., community centers, heart sports groups outdoors, etc.)
- Home visits to patients
- Other: ___

**A5.** In which federal state do you primarily work in the care of cardiology patients?
If you work equally at multiple locations, select multiple options.

- Burgenland
- Carinthia
- Lower Austria
- Upper Austria
- Salzburg
- Styria
- Tyrol
- Vorarlberg
- Vienna
- No answer

**Part B: Physical Activity**

**B1.** To promote and maintain health, adults should:

- Engage in 150 to 300 minutes (2 ½ to 5 hours) of moderate-intensity endurance-oriented activity per week, **or**
- 75 to 150 minutes (1 ¼ to 2 ½ hours) of higher-intensity endurance-oriented activity per week, **or**
- A combination of moderate and high-intensity endurance-oriented activities.

Do you personally meet this recommendation?

- Yes
- No
- No answer

**B2.** How much time per week do you currently spend on endurance-oriented activity?
(Please state the minutes per week.)

**B3.** Additionally, adults should perform muscle-strengthening exercises involving all major muscle groups on two or more days a week.

Do you personally meet this recommendation?

- Yes
- No
- No answer

**B4.** On how many days per week do you currently perform muscle-strengthening exercises?

**Part C: Affinity for Digital Technology**

**C1.** The following questions address your personal opinion on digital technologies and your experience with these technologies.

Digital technologies include devices/applications such as:

- Computers, laptops, mobile phones, smartphones, iPhones, tablets, iPads
- Email, Internet
- Smartwatches, pedometers, activity trackers (e.g., Fitbit)
- Heart rate monitors, wearable devices, power meters
- Apps for tracking, reminders, social networks (e.g., Strava, Facebook)

Please indicate how well the following statements apply to you by marking the appropriate box:

| **Statement** | **Fully applies** | **Mostly applies** | **Neutral** | **Rarely applies** | **Does not apply** |
| --- | --- | --- | --- | --- | --- |
| Digital technologies make things more complicated. |  |  |  |  |  |
| Digital technologies make my daily life easier. |  |  |  |  |  |

**Part D: Private Use of Digital Technologies**

**D1.** In the next questions, we would like to know whether you use digital technologies in your private daily life.

Do you use one or more of the following digital devices in your private daily life?
(Multiple answers possible)

- Smartphone
- Smartwatch
- Pedometer
- Wristwatch with heart rate monitoring
- Chest strap with heart rate monitoring
- Performance measurement (e.g., power meter on a bicycle)
- None
- Other: ___

**D2.** Do you use one or more digital applications in your private daily life to record, plan, or share your activities?
(e.g., fitness apps/fitness communities such as Freeletics, Strava, Outdooractive, ...)

- Yes
- No
- No answer

**D3.** Please name the applications you use most frequently: ___

**Part E: Work-Related Use of Digital Technologies**

**E1.** The following questions relate to the current use of digital technologies in your work with cardiology patients.

Please consider the following technologies:

- Apps
- Activity trackers and other wearables
- Online information services
- Tele-rehabilitation systems
- Digital patient information and management systems
- Applications based on artificial intelligence

Please only consider digital technologies that involve patient participation.

Do you recommend the use of digital technologies to your patients?

- Yes
- No

**E2.** Please briefly describe which digital technologies you recommend, for what purpose, and to which patients: ___

**E3.** If there are specific reasons why you do not recommend digital technologies, please describe them here: ___

**E4.** Do you use digital technologies in your work with cardiology patients?

- Yes
- No

**E5.** Which of the following digital technologies do you regularly use or offer as part of cardiological rehabilitation?
(Multiple answers possible)

- Activity trackers
- Pedometers
- Smartwatches
- Wristwatch with heart rate monitoring
- Chest strap with heart rate monitoring
- Podcasts
- Online information
- Apps
- None
- Other: ___

**E6.** Please name the apps you use with your patients: ___

**E7.** If there are specific reasons why digital technologies are not used, please describe them here: ___

**E8.** Are there digital technologies you only offer to certain patients?
If yes, check the relevant options and briefly describe the target group or indications:

- Activity trackers (e.g., pedometers): ___
- Wristwatch with heart rate monitoring: ___
- Chest strap with heart rate monitoring: ___
- Online information (e.g., training videos): ___
- Apps: ___
- No: ___
- Other: ___

**E9.** Have you previously used digital technologies with patients that you no longer use?

- Yes
- No

**E10.** Please briefly describe which digital technology(ies) you used and why they are no longer in use: ___

**E11.** Do you know if digital technologies are used by other colleagues in cardiological rehabilitation, for example, in specific rehabilitation facilities?

- Yes
- No

**E12.** Please briefly describe which digital technology(ies) are used by colleagues or in other rehabilitation facilities and for what purpose: ___

**Part F: Subjective Assessment**

**F1.** How would you describe your personal willingness to use digital technologies in your professional work with cardiology patients in general?

- Very willing
- Somewhat willing
- Neutral
- Somewhat unwilling
- Very unwilling

**Part G: Barriers to Use**

**G1.** The next section focuses on your assessment of barriers to using digital technologies in the care of cardiology patients.

Please consider the following technologies:

- Apps, activity trackers, and other wearables
- Online information services
- Tele-rehabilitation systems
- Digital patient information and management systems
- Applications based on artificial intelligence

Please only consider digital technologies that involve patient participation.

Which of the following aspects do you perceive as barriers to using digital technologies in the care of cardiology patients?

|  | **Fully applies** | **Mostly applies** | **Neutral** | **Rarely applies** | **Does not apply** | **Don’t know** |
| --- | --- | --- | --- | --- | --- | --- |
| Immature technology |  |  |  |  |  |  |
| Inaccurate measurements from wearables/sensors |  |  |  |  |  |  |
| Poor user-friendliness of devices/applications |  |  |  |  |  |  |
| Lack of technical competence among patients |  |  |  |  |  |  |
| Lack of technical competence among staff |  |  |  |  |  |  |

### **G2. Which of the following aspects do you perceive as a barrier to using digital technologies in the care of cardiology patients?**

**I perceive the following as barriers:**

|  | **Fully Agree** | **Partially Agree** | **Neutral** | **Partially Disagree** | **Fully Disagree** | **I Don’t Know** |
| --- | --- | --- | --- | --- | --- | --- |
| Low trust of medical staff in the technology (doubts about effectiveness) |  |  |  |  |  |  |
| Staff frustration with emerging difficulties |  |  |  |  |  |  |
| Increased workload for staff due to digital technology (e.g., additional steps required compared to analog work) |  |  |  |  |  |  |
| Possible data & information overload for staff |  |  |  |  |  |  |
| Staff fear of external control (monitoring) |  |  |  |  |  |  |
| Shift of decision-making from staff to technology (loss of control over treatment) |  |  |  |  |  |  |

### **G3. Which of the following aspects do you perceive as a barrier to using digital technologies in the care of cardiology patients?**

**I perceive the following as barriers:**

|  | **Fully Agree** | **Partially Agree** | **Neutral** | **Partially Disagree** | **Fully Disagree** | **I Don’t Know** |
| --- | --- | --- | --- | --- | --- | --- |
| Lack of / limited internet access for patients |  |  |  |  |  |  |
| Low trust of patients in the technology |  |  |  |  |  |  |
| Patient concerns about potential health risks |  |  |  |  |  |  |
| Patient concerns about their privacy |  |  |  |  |  |  |
| Additional costs for patients |  |  |  |  |  |  |

### **G4. Which of the following aspects do you perceive as a barrier to using digital technologies in the care of cardiology patients?**

**I perceive the following as barriers:**

|  | **Fully Agree** | **Partially Agree** | **Neutral** | **Partially Disagree** | **Fully Disagree** | **I Don’t Know** |
| --- | --- | --- | --- | --- | --- | --- |
| High acquisition costs for providers of rehabilitation or medical care |  |  |  |  |  |  |
| Additional ongoing costs per patient treatment for providers |  |  |  |  |  |  |
| Complicated billing process for providers with insurance companies |  |  |  |  |  |  |
| Digital applications are not covered by insurance providers |  |  |  |  |  |  |

**G5.** Are there other aspects that you believe hinder the use of digital technologies in your work with cardiology patients? Please list all aspects that come to mind: ___

**Part H: Potential Applications**

**H1.** We would like to know your opinion on important areas of application for digital technologies in the care of cardiology patients.

To what extent do the following aspects represent important areas of application for digital technologies?

|  | **Very important** | **Somewhat important** | **Neutral** | **Somewhat unimportant** | **Not important at all** | **Don’t know** |
| --- | --- | --- | --- | --- | --- | --- |
| Organization/scheduling |  |  |  |  |  |  |
| Documentation of measures |  |  |  |  |  |  |
| Creation of personalized treatment plans |  |  |  |  |  |  |
| Monitoring training outside the rehab facility |  |  |  |  |  |  |
| Training control |  |  |  |  |  |  |
| Offering tele-rehabilitation |  |  |  |  |  |  |
| Telemedicine care |  |  |  |  |  |  |
| Continuous biometric remote monitoring (telemonitoring) |  |  |  |  |  |  |
| Monitoring the work performed by staff |  |  |  |  |  |  |

**H2.** To what extent do the following aspects represent important areas of application for digital technologies?

|  | **Very important** | **Somewhat important** | **Neutral** | **Somewhat unimportant** | **Not important at all** | **Don’t know** |
| --- | --- | --- | --- | --- | --- | --- |
| Communication with patients |  |  |  |  |  |  |
| Patient education |  |  |  |  |  |  |
| Supporting patients in adhering to desired lifestyle changes in general |  |  |  |  |  |  |
| Supporting patients in adhering to desired changes in physical activity specifically |  |  |  |  |  |  |
| Monitoring the implementation of agreed-upon goals |  |  |  |  |  |  |
| Monitoring the course of disease in the short term |  |  |  |  |  |  |
| Monitoring the course of disease in the long term |  |  |  |  |  |  |
| Self-reporting/self-monitoring of results by patients |  |  |  |  |  |  |

**H3.** Are there other areas of application for digital technologies in the care of cardiology patients that you believe would be useful?

Assume that anything would be theoretically possible. Feel free to share your thoughts without restrictions: ___

**Part I: Decision-Influencing Factors**

**I1.** The next section is about various aspects that can influence the decision to use or not use digital technologies.

How important are the following aspects in your decision to use digital technologies?

|  | **Very important** | **Somewhat important** | **Neutral** | **Somewhat unimportant** | **Not important at all** | **Don’t know** |
| --- | --- | --- | --- | --- | --- | --- |
| Availability of IT support |  |  |  |  |  |  |
| Scientific evidence of the digital technology's reliability |  |  |  |  |  |  |
| Scientific evidence for the effectiveness of digital health interventions |  |  |  |  |  |  |
| Compatibility with the electronic health record (ELGA) |  |  |  |  |  |  |
| Compatibility with existing digital technologies |  |  |  |  |  |  |
| Personalizability for your own needs |  |  |  |  |  |  |
| Your own and colleagues’ prior experiences with digital technologies |  |  |  |  |  |  |
| Appearance/design of the digital technology |  |  |  |  |  |  |

### **I2. How important are the following aspects in your decision to use digital technologies?**

|  | **Very Important** | **Rather Important** | **Neutral** | **Rather Unimportant** | **Not Important at All** | **I Don’t Know** |
| --- | --- | --- | --- | --- | --- | --- |
| Ensuring uniform use of digital technology within the organization |  |  |  |  |  |  |
| Support from the leadership and management of your organization in transitioning to and using digital technology |  |  |  |  |  |  |
| Autonomy in your own workflows |  |  |  |  |  |  |
| Your involvement as an implementer in the decision-making process |  |  |  |  |  |  |
| Training opportunities provided through digital technologies |  |  |  |  |  |  |
| Liability concerns in the event of medical complications caused by technical errors |  |  |  |  |  |  |
| Proactive collaboration between health policy and medical associations |  |  |  |  |  |  |

### **I3. How important are the following aspects in your decision to use digital technologies?**

|  | **Very Important** | **Rather Important** | **Neutral** | **Rather Unimportant** | **Not Important at All** | **I Don’t Know** |
| --- | --- | --- | --- | --- | --- | --- |
| Personal/human contact between you and your patients |  |  |  |  |  |  |
| Age of the patients |  |  |  |  |  |  |
| Ensuring patient safety |  |  |  |  |  |  |
| Ensuring patient privacy |  |  |  |  |  |  |
| Familiarity with the digital technology |  |  |  |  |  |  |
| Endorsement of digital technologies by recognized institutions (e.g., health insurance providers, WHO, etc.) |  |  |  |  |  |  |
| Improving access to medical services and reaching underserved populations through digital technologies |  |  |  |  |  |  |

**I4.** Are there other important aspects you would consider in your decision-making process that have not yet been mentioned? Please list them here: ___

**Part J: Personal Demographic Data**

**J1.** Finally, we ask you to provide some information about yourself. Please select the answers that apply to you.

What gender do you identify with?

- Female
- Male
- Diverse
- No answer

**J2.** How old are you? ___

**J3.** What is your highest level of completed education?

- Compulsory schooling
- Apprenticeship with professional training
- Vocational or commercial school
- High school diploma (Matura or Abitur)
- Degree from a university or (applied) college
- Other: ___

**J4.** What professional/academic training have you completed?
(Multiple answers possible)

- Dietetics
- Occupational therapy
- Nutritional science
- Health and nursing care
- Speech therapy
- Medicine
- Medical assistant
- Administrative/office management
- Physiotherapy
- Psychology
- Psychosomatics
- Psychotherapy
- Social work
- Sports science
- Other: ___
